# Laboratory Practices and Incidence of Non-O157 Shiga Toxin–producing *Escherichia coli* Infections

**DOI:** 10.3201/eid1803.111358

**Published:** 2012-03

**Authors:** Kathleen A. Stigi, J. Kathryn MacDonald, Anthony A. Tellez-Marfin, Kathryn H. Lofy

**Affiliations:** Washington State Department of Health, Shoreline, Washington, USA

**Keywords:** Escherichia coli, Shiga-toxigenic Escherichia coli, Shiga toxin 2, Shiga toxin 1, Escherichia coli O157, hemolytic uremic syndrome, HUS, virulence factors, enterohemorrhagic E. coli, STEC, bacteria, Escherichia coli, E. coli

## Abstract

We surveyed laboratories in Washington State, USA, and found that increased use of Shiga toxin assays correlated with increased reported incidence of non-O157 Shiga toxin–producing *Escherichia coli* (STEC) infections during 2005–2010. Despite increased assay use, only half of processed stool specimens underwent Shiga toxin testing during 2010, suggesting substantial underdetection of non-O157 STEC infections.

Strains of Shiga toxin (Stx)–producing *Escherichia coli* (STEC) are differentiated by the O antigen on their outer membrane and are broadly classified as O157 or non-O157 STEC (*1**–**3*). The ability to produce Stx is a key virulence trait of STEC (*1**,**3**,**4*). STEC infections in humans often cause a self-limited diarrheal illness but can be complicated by hemorrhagic colitis or hemolytic uremic syndrome (*1*).

Unlike other *E. coli* strains, serogroup O157 isolates do not ferment sorbitol and are readily identified by culture, appearing colorless on sorbitol MacConkey agar (*1**,**2**,**4*). Both O157 and non-O157 STEC can be identified by detecting Stx with nonculture assays that became commercially available in the United States in 1995 (*2**,**4*). The Centers for Disease Control and Prevention (CDC) published formal STEC testing recommendations for clinical laboratories in 2009, advocating that all stool specimens submitted for routine bacterial pathogen testing be simultaneously cultured for O157 STEC and tested with a nonculture assay to detect Stx. Use of this testing protocol ensures timely identification of all STEC infections (*2**,**5*). Exclusive testing for Stx delays specific identification of O157 STEC and may impede prompt detection of common-source outbreaks (*2**–**4*).

Non-O157 STEC infection has been a nationally notifiable condition since 2000 (*5*). Although studies have documented the increased incidence of reported non-O157 STEC infections over the past decade, few have determined the proportion of laboratories that routinely test all submitted stool specimens for Stx and, to our knowledge, no study has quantified STEC testing practices by proportion of stool specimens processed for bacterial culture. Our objectives, therefore, were to quantify statewide STEC testing practice by proportion of stool specimens processed for bacterial culture and to determine the contribution of enhanced STEC testing practice to increased reported incidence of non-O157 STEC infections.

## The Study

**Figure 1:**
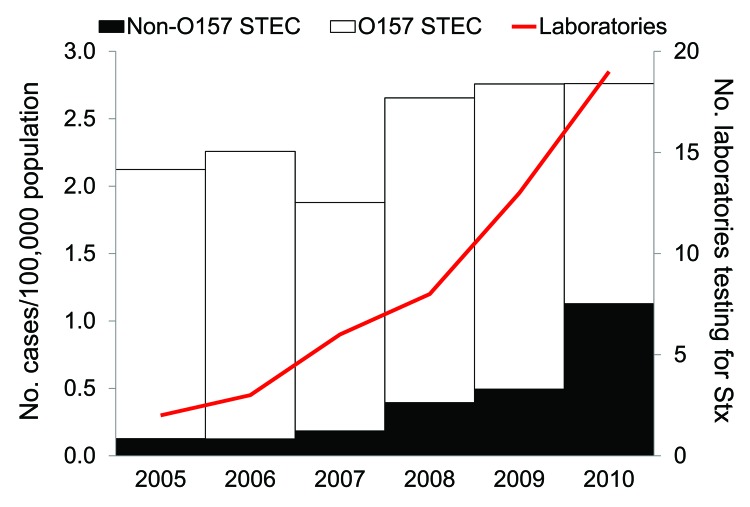
Rate of reported O157 and non-O157 Shiga toxin (Stx)–producing *Escherichia coli* (STEC) infections and number of laboratories performing Stx testing by year, Washington State, USA, 2005–2010.

Data for all confirmed STEC infections reported to the Washington State Department of Health (DOH) with illness onset during 2005–2010 were reviewed to determine incidence trends. Confirmed STEC is defined as the isolation of *E. coli* O157:H7 or an Stx-producing *E. coli* isolate from a clinical specimen. Of 945 cases reported to DOH during 2005–2010 (average annual incidence: 2.4 cases/100,000 population), 781 (83%) cases were O157 STEC and 164 (17%) cases were non-O157 STEC infections. The incidence of non-O157 STEC infections increased dramatically during the 6-year period, from 0.13/100,000 population and 6% of all reported STEC infections in 2005 to 1.13/100,000 population and 41% of all reported STEC infections in 2010 (Figure 1). Four serogroups accounted for >80% of non-O157 STEC cases: O26 (48%), O103 (18%), O121 (12%), and O111 (5%).

Using data from the Washington State DOH Office of Laboratory Quality Assurance, we identified 74 clinical microbiology laboratories in the state. To assess statewide STEC laboratory testing practices, we developed an online survey and distributed it to microbiology laboratory supervisors at these 74 laboratories during January 2011. Seventeen laboratories reported that all stool specimens are forwarded to a reference laboratory for testing. These 17 laboratories were excluded from the survey sample; however, all indicated reference laboratories were within the state and among the remaining sample (n = 57). Follow-up was completed by email and telephone until a 100% response rate was achieved. The survey requested data on annual number of stool specimens processed for bacterial culture, current protocol for processing stool specimens submitted for routine enteric pathogen testing, and motivations and barriers toward the implementation of Stx testing. If Stx testing was reported, we requested implementation date. Laboratory supervisors were asked to indicate a range for the number of stool specimens processed for bacterial culture at their laboratory during 2010. The 32 laboratories (56%) that reported >300 specimens were asked to specify the quantity. For the 25 laboratories that reported <300 specimens, a midpoint of the range was assigned. All data were analyzed by using SAS for Windows, version 9.2 (SAS Institute, Inc., Cary, NC, USA).

Fifty-seven laboratories in Washington State collectively processed an estimated 71,000 stool specimens for bacterial culture in 2010; the number of specimens ranged from 61 to 6,017 specimens per laboratory (median 570). The 10 (18%) largest laboratories processed 51% of the total annual specimens, while the 25 (44%) smallest laboratories processed only 5% of the total annual specimens.

**Figure 2:**
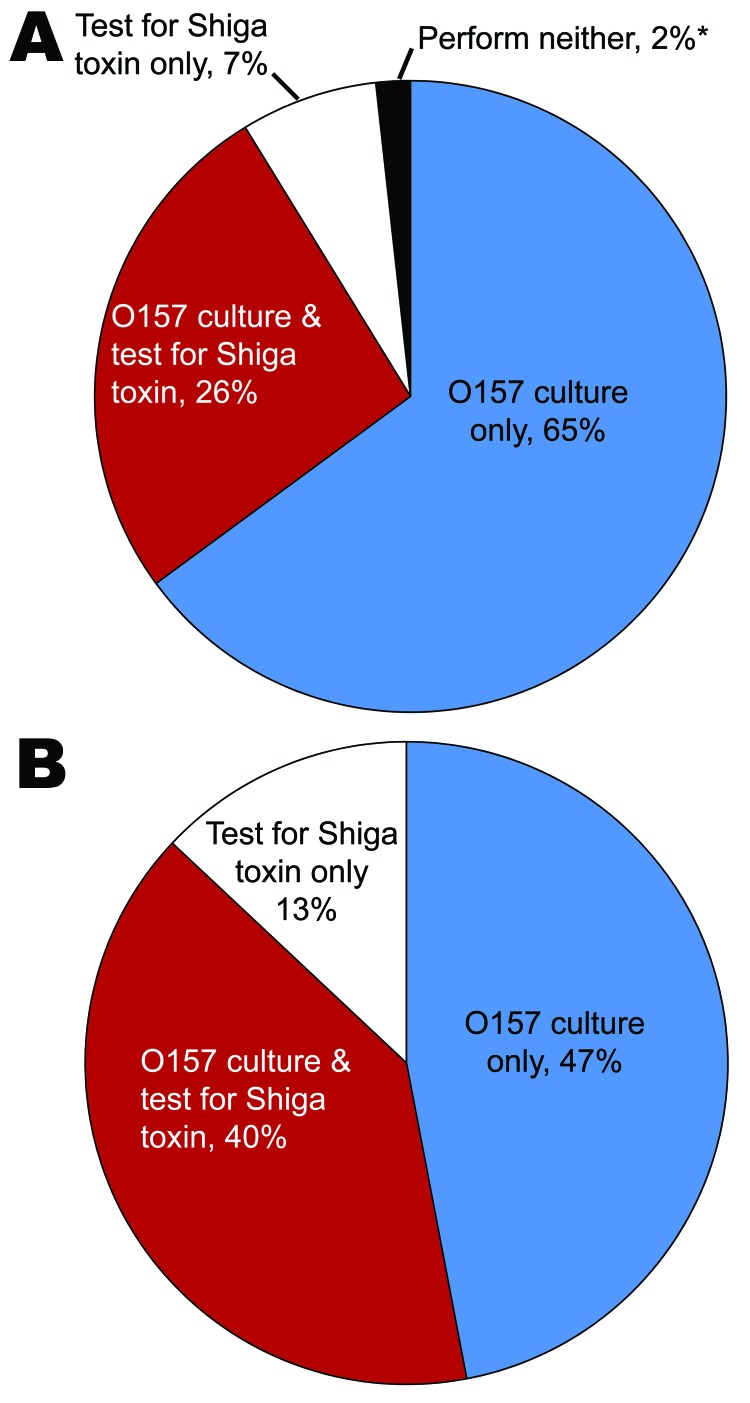
Routine clinical laboratory practice to detect Shiga toxin (Stx)–producing *Escherichia coli* (STEC) by proportion of laboratories (A) and proportion of annually processed stool specimens (B), Washington, USA, 2010. *One laboratory reported use of neither method but represented <0.02% of annually processed specimens.

The following results quantify reported routine enteric pathogen testing protocols for all stool specimen submissions. Of 57 laboratories, 56 (98%) performed routine STEC testing on all submitted stool specimens, either by culture, by detecting the presence of Stx with nonculture assays, or both. Fifteen (26%) reported simultaneous culture for O157 STEC and Stx testing, 37 (65%) cultured for O157 STEC exclusively, and 4 (7%) tested for Stx exclusively (Figure 2, panel A). Combining the number of processed specimens and testing protocol for each laboratory, we estimated that 40% of stool specimens in Washington State were cultured for O157 STEC and tested for Stx, 47% were cultured for O157 STEC exclusively, and 13% were tested for Stx exclusively (Figure 2, panel B).

Of the 19 laboratories that tested for Stx, 11 (58%) implemented testing in 2009 or 2010 (Figure 1). When asked about motivations to implement Stx testing, laboratories most commonly reported CDC recommendations and the desire to detect non-O157 STEC infections. The most commonly reported barriers were cost, procedural change, and staffing constraints.

## Conclusions

During 2005 through 2010, the number of laboratories in Washington State that tested for Stx increased from 2 (4%) in 2005 to 19 (33%) in 2010, and the incidence of reported non-O157 STEC infections increased from 8 cases (0.13/100,000 population) in 2005 to 76 cases (1.13/100,000) in 2010. The most dramatic increase in reported non-O157 STEC infections occurred between 2008 and 2010, during which time incidence increased nearly 3-fold, from 26 cases (0.39/100,000) in 2008 to 76 cases (1.13/100,000) in 2010. This increase in reported incidence occurred at the same time during which most laboratories that test for Stx (11, or 58%) implemented testing (Figure 1). This suggests the increase in the reported incidence of non-O157 STEC is likely caused by changes in testing practice.

Despite the increased use of Stx testing, 37 (65%) of the laboratories in Washington State that processed nearly half (47%) of the stool cultures in the state during 2010 cultured for O157 STEC exclusively and, therefore, could not detect non-O157 STEC. Had all specimens been tested for both O157 and non-O157 STEC, we estimate that the incidence of non-O157 STEC would have been 2.12/100,000 population (60% of all reported STEC infections) in 2010, rather than the reported 1.13/100,000 (40% of all reported STEC infections).

Enhanced detection and reporting of STEC infections will likely increase workloads for local communicable disease investigators and public health laboratories during a time when funding for public health is limited. At the local level, every reported STEC infection requires an epidemiologic investigation, while additional detection of Stx at clinical laboratories will increase submission volume at public health laboratories.

National studies have found that non-O157 STEC infections are clinically indistinguishable from O157 STEC infections, with comparable hemolytic uremic syndrome attack rates (*5**–**7*). The potential virulence of non-O157 STEC infections underscores the need for enhanced laboratory testing and epidemiologic research. To encourage adherence to STEC testing recommendations, healthcare providers should request Stx testing if it is not routinely performed at their laboratory. Public health professionals and epidemiologists are encouraged to assess STEC testing practices to correctly interpret incidence trends and make clinical comparisons.
